# Multiple Dissociations Between Comorbid Depression and Anxiety on Reward and Punishment Processing: Evidence From Computationally Informed EEG

**DOI:** 10.1162/cpsy_a_00024

**Published:** 2019-01

**Authors:** James F. Cavanagh, Andrew W. Bismark, Michael J. Frank, John J. B. Allen

**Affiliations:** Department of Psychology, University of New Mexico, Albuquerque, New Mexico, USA; VA San Diego Healthcare System, San Diego, California, USA; Department of Cognitive, Linguistic, and Psychological Sciences, Brown University, Providence, Rhode Island, USA; Department of Psychology, University of Arizona, Tucson, Arizona, USA

**Keywords:** depression, anxiety, FRN, Rew-P, reinforcement learning, computational psychiatry

## Abstract

In this report, we provide the first evidence that mood and anxiety dimensions are associated with unique aspects of EEG responses to reward and punishment, respectively. We reanalyzed data from our prior publication of a categorical depiction of depression to address more sophisticated dimensional hypotheses. Highly symptomatic depressed individuals (*N* = 46) completed a probabilistic learning task with concurrent EEG. Measures of anxiety and depression symptomatology were significantly correlated with each other; however, only anxiety predicted better avoidance learning due to a tighter coupling of negative prediction error signaling with punishment-specific EEG features. In contrast, depression predicted a smaller reward-related EEG feature, but this did not affect prediction error coupling or the ability to learn from reward. We suggest that this reward-related alteration reflects motivational or hedonic aspects of reward and not a diminishment in the ability to represent the information content of reinforcements. These findings compel further research into the domain-specific neural systems underlying dimensional aspects of psychiatric disease.

## INTRODUCTION

The shift from a categorical to a dimensional depiction of psychiatric disorders has led to a reassessment of prior knowledge (Insel et al., 2010). In line with this new perspective, we present a reassessment of a prior categorical depiction of depression to address more sophisticated dimensional hypotheses.

In our 2011 article (Cavanagh, Bismark, Frank, & Allen, 2011), we described how individuals with current or past major depressive disorder (MDD) had increased neural signals to punishment, leading to a punishment learning bias in a reinforcement learning task. The present report provides a more detailed analysis of these data, including computational modeling and source estimation. Recent advancements in the field motivated us to examine the unique, dimensional influences of depression or anxiety on reward versus punishment processing. We believe that the findings in this current report present a more complete description of the original findings, while demonstrating powerful new methods for dissociating the influence of latent cognitive and affective states via EEG measures of reward and punishment processing.

Although symptoms of depression and anxiety are highly comorbid, there are good reasons to think that they should be separable at the level of neural systems (Drysdale et al., 2017). Anxiety is associated with hypervigilance in amygdala and salience-related circuits (Fox et al., 2015; Shackman et al., 2011), particularly to threats and punishments (Cavanagh & Shackman, 2014; Moser, Moran, Schroder, Donnellan, & Yeung, 2013; Tovote, Fadok, & Lüthi, 2015). Depression may be specifically defined by processes influenced by the cardinal symptoms of sadness and anhedonia, such as hedonic capacity and reward responsivity (Admon & Pizzagalli, 2015; Keedwell, Andrew, Williams, Brammer, & Phillips, 2005; Romer Thomsen, Whybrow, & Kringelbach, 2015). Although there is more to anxiety and depression than punishment and reward responsiveness, the computational roles of these processes are well defined, and EEG offers a chance to assess neural signals that are sensitive and sometimes specific to each process.

As a category, individuals with MDD are characterized by a negativity bias and hypersensitivity to punishment (Beck, 1976; Elliott et al., 1996; Elliott, Sahakian, Herrod, Robbins, & Paykel, 1997; Eshel & Roiser, 2010). The feedback-related negativity (FRN) is a theta-band dominant EEG feature that is sensitively (but not specifically) elicited by punishments and is enhanced by worse-than-expected outcomes (Cavanagh, Frank, Klein, & Allen, 2010; Chase, Swainson, Durham, Benham, & Cools, 2010; Ichikawa, Siegle, Dombrovski, & Ohira, 2010). Larger FRNs and theta power following punishments have been observed in MDD participants (Cavanagh, Bismark et al., 2011; Tucker, Luu, Frishkoff, Quiring, & Poulsen, 2003; Webb et al., 2016), and our prior findings (Cavanagh, Bismark et al., 2011) presented evidence that these enhanced signals were related to a better ability to avoid stimuli previously associated with punishment. More recent findings in this field have described how FRN and theta power are reliably enhanced in anxiety (Cavanagh & Shackman, 2014; Moser et al., 2013; Weinberg, Olvet, & Hajcak, 2010), fitting with a conceptualization that they reflect a transdiagnostic marker of threat sensitivity (Riesel, Goldhahn, & Kathmann, 2017; Weinberg et al., 2016). In sum, emerging evidence suggests that larger error signals in this categorical sample may not be due to the diagnostic label of depression per se but rather may be more sensitive to comorbid anxiety.

A different EEG feature may be specifically affected by depression. The reward positivity (Rew-P) is a delta-band dominant EEG feature that is sensitively and specifically elicited by rewards and is enhanced by better-than-expected outcomes (Bernat, Nelson, Steele, Gehring, & Patrick, 2011; Cavanagh, 2015; Holroyd, Pakzad-Vaezi, & Krigolson, 2008). Depression is associated with a smaller Rew-P (Weinberg & Shankman, 2017) and delta-band response (Nelson et al., 2018), fitting with a conceptualization that this signal reflects reward sensitivity, a construct that would be most directly relevant to a depressive, not anxious, symptomatology (Nusslock & Alloy, 2017; Proudfit, 2015; Proudfit, Bress, Foti, Kujawa, & Klein, 2015).

These conceptual dissociations between possible neural biomarkers of depression and anxiety motivate the need for paradigms that can test this potentially important dissociation. If anxiety is more sensitive to punishment-related FRN/theta activity and depression is more sensitive to reward-related Rew-P/delta activity, then the unique variance in these brain activities should be assessed. The majority of the literature on the FRN and Rew-P (including many in psychiatric samples) interprets these in the context of a single reward-minus-punishment difference wave. A difference wave approach not only conflates the variance of punishment- and reward-related signals but confuses the information content of the individual signals and violates the assumption of pure insertion that underlies the logic of cognitive subtraction. The conflation of variance between control-related FRN/theta and reward-related Rew-P/delta signals is similar to that between depression and anxiety in the dimensional assessment of MDD. In this report, we provide the first evidence that mood and anxiety dimensions are associated with *unique* aspects of EEG responses to reward and punishment, respectively.

## MATERIAL AND METHODS

### Participants

All participants provided written informed consent that was approved by the University of Arizona. Participants were recruited from introductory psychology classes based on mass survey scores of the Beck Depression Inventory (BDI). Recruitment criteria included (a) age 18–25 years, (b) no history of head trauma or seizures, and (c) no current psychoactive medication use. Control participants (CTL: *N* = 75, 40 female) had stable low BDI (<7) between mass survey and experimental assessment (2–14 weeks), no self-reported history of MDD, and no self-reported symptoms indicating the possibility of an Axis I disorder as indicated by computerized self-report completion of the Electronic Mini International Neuropsychological Interview (eMINI).

Participants in the high depressive symptomatology group (DEP) needed to have a stable high BDI (≥13) between mass testing and experimental assessment. A total of 46 (34 female) participants met these criteria. Individuals from the DEP group were invited to participate in a paid ($20/hour) Structured Clinical Interview for Depression, and the individuals fell into four near-equivalent subgroups: *n* = 14 declined the interview, *n* = 9 did not meet criteria for MDD, *n* = 12 met criteria for past MDD, and *n* = 11 met criteria for current MDD. The prior report on these data (Cavanagh, Bismark et al., 2011) used a diagnostic classes approach, utilizing the current and past MDD individuals (*N* = 21). This current report aimed to use a dimensional approach so all 46 participants were included.

In the experimental visit, all participants first completed the BDI as well as the Spielberger Trait Anxiety Inventory (TAI). Based on recent decompositions of the factor structure of the BDI (Vanheule, Desmet, Groenvynck, Rosseel, & Fontaine, 2008), the weighted average of the cognitive (Items 2, 3, 6, 8, 9, 14) and affective (Items 4, 10, 12) subscales were used to probe the specific facets of depressive symptomatology that were expected to be sensitive to reinforcement signals. Given the highly restricted range of symptomatology in the CTL group, the DEP and CTL groups were examined as representations of separate populations.

### Task

**Figure 1.**
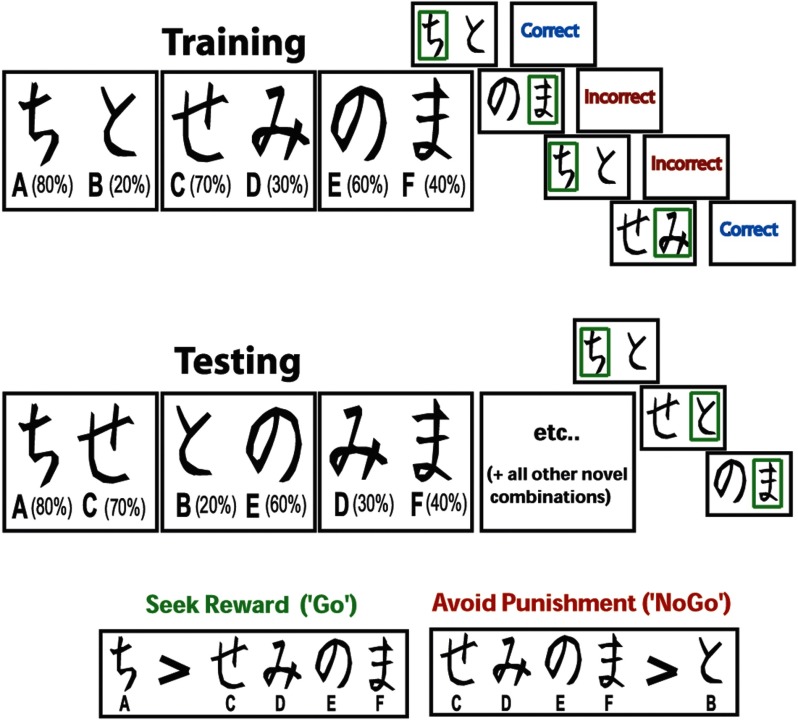
**Probabilistic learning task.** During training, each pair is presented separately. Participants have to select one of the two stimuli, slowly integrating “correct” and “incorrect” feedback (each stimulus has a unique probabilistic chance of being correct) to maximize their accuracy. The EEG activities reported here were taken following these feedbacks. During the testing phase, each stimulus is paired with all other stimuli, and participants must choose the best one, without the aid of feedback. Measures of reward and punishment learning are taken from the test phase, hypothesized to reflect the operations of a slow, probabilistic integrative system during training. Note that the letter and percentage are not presented to the participant, nor are the green boxes surrounding the choice.

Participants performed a probabilistic learning task using different pseudo-randomly assigned character sets. This task included a forced choice training phase followed by a subsequent testing phase (Frank, Seeberger, & O’Reilly, 2004), as shown in Figure 1. During the training phase, the participants were presented with three stimulus pairs, where each stimulus was a Japanese Hiragana character associated with a different probabilistic chance of receiving “correct” or “incorrect” feedback. These stimulus pairs (and their probabilities of reward) were termed A/B (80%/20%), C/D (70%/30%), and E/F (60%/40%). All training trials began with a jittered intertrial interval (ITI) between 300 and 700 ms. The stimuli then appeared for a maximum of 4,000 ms and disappeared immediately after the choice was made. If the participant failed to make a choice within the 4,000 ms, “no response detected” was presented. Following a button press, either “correct” or “incorrect” feedback was presented for 500 ms (jittered between 50 and 100 ms postresponse).

During the testing phase, all possible stimulus pairs were presented eight times (120 trials total). Trials in the test phase began with an ITI of 500 ms. Stimuli were presented for a maximum of 4,000 ms and disappeared as soon as a choice was made. No feedback was provided in the testing phase. Reward seeking (“Go learning”) was defined as the accuracy of choosing A over C, D, E, and F (i.e., seeking A), whereas punishment avoidance, or “NoGo learning,” was defined as the accuracy of choosing C, D, E, and F over B (i.e., avoiding B). EEG signals were taken from the training phase (responses to feedback during learning), and behavioral indices of learning were taken from the testing phase. As in previous reports, we contrast Go minus NoGo measures for a single measure of biased learning. This analytic strategy allows an assessment of how the neural processing of feedback during learning relates to value-based decision-making at a later point in time.

### Computational Modeling

State-action values were estimated for each stimulus type, and a softmax choice function was used to predict the most likely action on each trial. State-action values (*Q*values) were updated according to the delta learning rule with a learning rate (α) scaling the prediction error (δ),

$$Q_{t}=Q_{t-1}+\alpha (\delta ),$$ (1)

where prediction errors were calculated as the difference between reinforcements (*R*) and *Q* values,

δ=r−Q, (2)

and reinforcements were from a set of 0, 1,

r∈(0,1). (3)

The probability of action selection was predicted using a softmax logistic function with a free parameter for gain adjustment to select the highest-value option (β, also termed behavioral consistency or inverse temperature):

$$p(Q_{\text{selected}})=\exp(\beta \cdot Q_{\text{selected}})/\sum_{\text{all}}\exp(\beta \cdot Q_{\text{all}}).$$ (4)

The computationally derived prediction error (PE; Equation 2) was used as a single-trial regressor in EEG analyses. Based on prior findings, we anticipated that a model with separate learning rates for gain and loss would best account for the data (Cavanagh, Frank, & Allen, 2011; Frank, Moustafa, Haughey, Curran, & Hutchison, 2007). Two competing models were formally compared (common learning rate vs. gain and loss learning rates), and PEs from the best-fitting model were used as regressors for EEG analyses. The probabilities of action selection (Equation 4) were used to compute the log likelihood estimate (LLE) of the participant having chosen that set of responses for a given set of parameters. The parameters that produced the maximum LLE were found using the Nelder–Mead simplex method, a standard hill-climbing search algorithm (implemented with Matlab function fminsearch.m).

All models used the best-fitting outcome of 10 different starting points (using Matlab function rmsearch.m). Following convention (Daw, 2011), all learning rates were constrained to remain between 0 and 1, all softmax gain parameters were constrained to be above zero, and all *Q* values began at 0.5 for equiprobable initial selection. Characterizations of model fits were computed as pseudo-*R*^2^ statistics: (LLE − chance)/chance (Camerer & Ho, 1999). For model comparison, more complex models were penalized by computing the Akaike information criterion (AIC) for each subject. The likelihood ratio test (2 ⋅ LLE[model1] − LLE[model2]) and associated *p* value from the chi-square cumulative density function (Matlab function chi2csf.m) was used to investigate the relative performance of competing models.

### Electrophysiological Recording and Processing

Scalp voltage was measured using 64 Ag/AgCl electrodes using a Synamps^2^ system (band-pass filter 0.5–100 Hz, 500 Hz sampling rate, impedances < 10 kΩ; the online reference was a single channel placed between Cz and CPz). All data were epoched around the feedback onset (−2,000 to 2,000 ms), from which the associated feedback-locked responses were isolated. “Cerebellar” leads were then removed from the data structure, yielding 60 total EEG channels and two mastoids that were submitted to preprocessing. Bad channels and bad epochs were identified using a conjunction of the FASTER algorithm (Nolan, Whelan, & Reilly, 2010) and pop_rejchan from EEGlab (Delorme & Makeig, 2004) and were subsequently interpolated and rejected, respectively. Eye blinks were removed following ICA. Data were then referenced to averaged mastoids.

Event-related potential (ERP) data were filtered between 0.1 and 20 Hz and baseline corrected to the average activity from −200 to 0 ms prefeedback. The time window for punishment-locked FRN ERP activity is identical to our prior report from this data set and was computed as the difference between the P3 (376 ms) and the N2 (276 ms) at the FCz electrode. The selection of this peak and trough helps to capture the underlying phase-locked theta-band dynamic (a half-cycle with 100 ms difference = 5 Hz). The reward-locked Rew-P ERP activity was quantified as the average activity from 250 to 350 ms at FCz, as in our prior report on that same component (Cavanagh, 2015).

Time–frequency measures were computed by multiplying the fast Fourier transformed (FFT) power spectrum of single-trial EEG data with the FFT power spectrum of a set of complex Morlet wavelets defined as a Gaussian-windowed complex sine wave: *e*^*i*2π*tf*^ *e*^−*t*^2^/(2*x*σ^2^)^, where *t* is time, *f* is frequency (which increased from 1–50 Hz in 50 logarithmically spaced steps), and the widths (or “cycles”) of frequency bands were set to increase from 3/(2π*f*) to 10/(2π*f*) as frequency increased. Then, the time series was recovered by computing the inverse FFT. Averaged power was normalized by conversion to a decibel (dB) scale (10 ⋅ log10[power(*t*)/power(baseline)]) from a common cross-condition averaged baseline of −300 to −200 ms, allowing a direct comparison of effects across frequency bands.

The time–frequency regions of interest (tf-ROI) were broadly expected to be at the FCz electrode in the theta band following punishment and in the delta band following reward; here we defined the precise windows based on the CTL group and used these windows for tf-ROI analysis within the DEP group. The punishment tf-ROI was 4–8 Hz from 200 to 400 ms, and the reward tf-ROI was 2–3 Hz from 200 to 400 ms. This out-of-sample procedure effectively protects against “double dipping” (Kriegeskorte, Simmons, Bellgowan, & Baker, 2009) and, combined with the very strong a priori hypotheses, adequately protects against multiple comparisons. As such, the statistical contrasts (correlation with symptoms) were not corrected for multiple comparisons; rather, they are shown with a cluster threshold of 500 pixels to demonstrate the effectiveness of the tf-ROI in capturing the individual differences of interest.

### Source Estimation

The sLORETA toolbox was used for source estimation of these ERP time windows (Pascual-Marqui, 2002). The sLORETA package uses minimum norm estimation to estimate the smoothest distribution, or lowest estimation error within voxels restricted to cortical tissue. To discover the sources of these different ERP time windows with effective contrasts, the upper and lower tertiles of PEs were contrasted within the CTL group specifically. This contrast is ideal, as it highlights the computational function specific to each condition and does not confuse variance between the conditions. For punishment, the null hypothesis test took the form of High PE P3 − High PE N2 = Low PE P3 − Low PE N2. For reward, this was tested as High PE Rew-P = Low PE Rew-P. Within the DEP group, correlations were tested for the influence of anxiety (P3 − N2 vs. anxiety) and the influence of depression (Rew-P vs. depression). For clarity, we performed this test and corresponding topographical correlations within five different 50 ms time windows leading up to and following the peak of the Rew-P. Subject-wise normalization was used for all contrasts and the nonparametric statistical package was used to test the outcomes versus 5,000 permutations. Cortical maps show *t* test or correlation coefficient outputs. Like any imaging method, sLORETA reveals a broad array of cortical activities associated with the contrast. Here we are not testing for a full depiction of brain areas associated with this activation; we are testing the more limited hypothesis that the midline structures associated with PE signaling in the FRN and Rew-P are different from each other and that different symptomatology affects different midline cortical areas for different outcomes. We report the outputs of sLORETA contrasts at the appropriately scaled level of Brodmann areas (BAs).

### Statistical Analyses

The hypotheses were that anxiety would specifically affect punishment-related processing (FRN, theta, NoGo learning) and depression would specifically affect reward-related processing (Rew-P and delta). While Go performance is used as our measure of reward learning, we were agnostic if depression would affect Go learning given our previously observed insensitivity of Rew-P to performance (Cavanagh, 2015) and prior null effects in the literature (Chase, Frank et al., 2010). Statistical analyses used Spearman’s correlations and the difference between correlations utilized Meng’s *z* test for correlated coefficients (Meng, Rosenthal, & Rubin, 1992). Because model parameters were nonlinearly distributed, nonparametric Mann–Whitney *U* tests were used. For clarity, all negative (punishment) PEs are presented as the absolute value (|−PE|). We also verified that all conclusions were maintained after assessment of unique variance in depression and anxiety, which were computed as the residual of each measure after accounting for the correlation between the two measures.

## RESULTS

### Behavior

**Figure 2.**
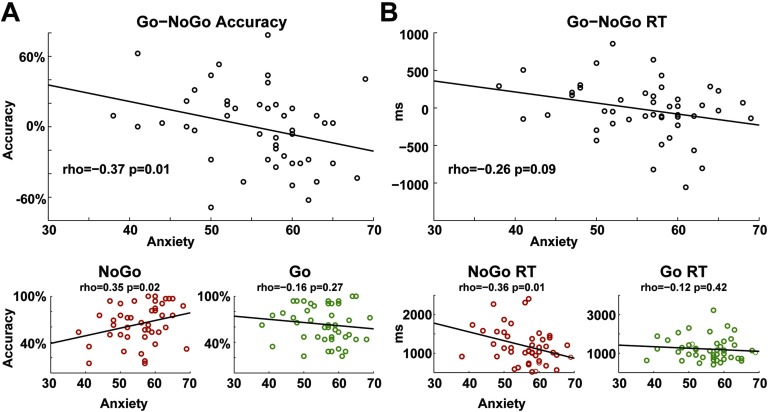
**Within the high depressive symptomatology group, anxiety predicted the bias to learn from punishment.** A) Anxiety correlated with the bias to learn more from NoGo than Go conditions; this effect was specific to the NoGo condition. B) Anxiety was related to nominally faster reaction times (RTs) for NoGo versus Go conditions, again entirely due to faster RTs on NoGo but not Go conditions. Together, these findings suggest that greater levels of anxiety were specifically related to a punishment learning bias.

There were no differences between DEP and CTL groups in any direct performance measures (see Table 1). Within the DEP group, measures of anxiety and depression symptomatology were significantly correlated with each other (ρ = 0.56, *p* < 0.01); however, only anxiety was related to valenced learning biases (Figure 2).

**Table 1.** Control (CTL) and depression (DEP) participant demographics, symptom scores, task performance, and best-fitting model parameters

	**CTL**	**DEP**	**Statistic and *p* value**
Sex			χ^2^ = 5.08, *p* = 0.02
Male	35	12	
Female	40	34	
Age (years)	18.97 (1.22)	18.74 (1.14)	*t* = 1.05, *p* = 0.30
BDI	1.73 (1.65)	22.22 (4.90)	*t* = 33.32, *p* < 0.01
TAI	31.05 (5.49)	55.76 (7.08)	*t* = 21.49, *p* < 0.01
No. trials	221 (115)	256 (110)	*t* = 1.61, *p* = 0.11
Train accuracy (%)	66 (9)	65 (9)	*t* = 0.52, *p* = 0.60
Test accuracy (%)	65 (11)	66 (12)	*t* = 0.34, *p* = 0.74
A > B accuracy (%)	78 (28)	78 (26)	*t* = 0.01, *p* = 0.99
Go accuracy (%)	64 (23)	63 (23)	*t* = 0.06, *p* = 0.95
NoGo accuracy (%)	63 (23)	64 (24)	*t* = 0.37, *p* = 0.71
Gain learning rate	0.26 (0.42)	0.11 (0.32)	*z* = 2.67, *p* = 0.008
Loss learning rate	0.06 (0.26)	0.01 (0.11)	*z* = 1.74, *p* = 0.08
Softmax gain	3.72 (4.38)	5.12 (8.76)	*z* = −1.58, *p* = 0.11

*Note*. All data are mean ± *SD*, except model parameters, which are median ± interquartile range. All *t* test *df* = 119. Aside from symptomatology, only sex differences and gain learning rate differentiated the groups. BDI = Beck Depression Inventory. TAI = Trait Anxiety Inventory.

Anxiety predicted the total learning bias (Go minus NoGo) in favor of more NoGo learning (ρ = −0.37, *p* = 0.01); it predicted more NoGo learning specifically (ρ = 0.35, *p* = 0.02) but not Go learning (ρ = −0.16, *p* = 0.27). Depressive symptoms did not predict learning accuracies (|ρ| < 0.08), and both the total bias and NoGo-specific correlations were specific to anxiety and not depression (Go–NoGo: Meng’s *z*= 2.30, *p* = 0.01; NoGo: Meng’s *z* = 1.94, *p* = 0.03). Response time showed similar trends, with anxiety predicting nonsignificantly faster reaction times (RTs) for Go minus NoGo (ρ = −0.26, *p* = 0.09) but significantly faster NoGo responding (ρ = −0.36, *p* = 0.01) yet no influence on Go RTs (ρ = −0.12, *p* = 0.42). Depressive symptoms did not predict any RTs (|ρ| < 0.06), and both the total bias and NoGo-specific correlations were specific to anxiety and not depression (Go–NoGo: Meng’s *z* = 1.6, *p* = 0.06; NoGo: Meng’s *z* = 2.17, *p* = 0.02). There were no significant correlations for depression or anxiety on performance within the CTL group. In sum, within the DEP group, individuals who were more anxious were better at learning to avoid stimuli previously associated with punishment, and they made these choices faster, suggesting greater decision confidence.

The model with separate learning rates for gain and loss fit the data better than a single learning rate (pseudo-*R*^2^: 18.45% vs. 15.63%; AIC: 280 vs. 287; LLE difference = 8.17; likelihood ratio test, *p* = 0.0043). The DEP group had a significantly lower learning rate for gain than the CTL group (Table 1). Within the DEP group, neither self-reported anxiety nor depression correlated with model parameters (|ρ| < 0.15, *p* > 0.31), which is surprising, because the relationship between greater anxiety and more punishment learning suggests that anxiety boosts some aspect of loss sensitivity.

### Event-Related Potentials

**Figure 3.**
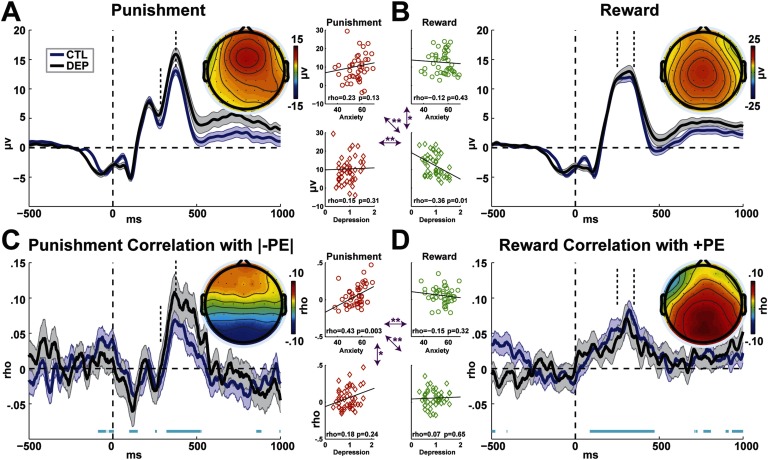
**Event-related potentials (ERPs) time locked to incorrect (red) and correct (green) feedback at electrode FCz.** A) Punishers (incorrect feedback) led to a N2–P3 complex, identified with vertical dashed lines. The P3–N2 difference was not different between groups, and against predictions, it did not have a strong correlation with anxiety (all scatterplots show relationships within the DEP group). B) Rewards (correct feedback) led to a reward positivity (Rew-P), identified with vertical dashed lines. The Rew-P did not differ between groups, but depression predicted a strong diminishment in amplitude; this relationship was differentiated from all other symptom–ERP correlations (magenta arrows). C) Correlation between single-trial absolute negative prediction error (|−PE|) and EEG activity, yielding a strong relationship in the same temporal domain as the P3–N2 complex. The average correlation between the P3 and N2 time points was significantly related to anxiety; this relationship was differentiated from all other correlations between symptomatology and PE–EEG coupling (magenta arrows). D) Correlation between single-trial positive prediction error (+PE) and EEG activity, yielding a strong relationship in the same temporal domain as the Rew-P. There were no relationships between depression and PE–EEG coupling. Horizontal cyan bars in C and D show significant *t* test outcomes from zero (uncorrected), demonstrating that PE–EEG coupling is robust in these previously identified component-specific time windows. All topoplots are raw values from the identified time windows. *p < 0.05. **p < 0.01.

There was no difference between DEP and CTL groups in FRN or Rew-P amplitudes (*t* < 1). While FRN amplitude was positively related to anxiety, it was nonsignificant (ρ = 0.23, *p* = 0.13; Figure 3A). However, Rew-P amplitude was significantly negatively correlated with depression (ρ = −0.36, *p* = 0.01; Figure 3B). None of the other conditions were significantly related to depression or anxiety, but the strong relationship between depression and Rew-P was significantly different than all other correlations (Meng’s *z* > 1.75, *p* < 0.04).

### EEG–PE Correlations

There was no difference between DEP and CTL groups in the relationship between EEG activity and PE within the a priori time windows (*t* < 1.24), hereinafter broadly termed EEG–PE coupling. Anxiety strongly correlated with the degree to which an individual’s |−PE| was reflected in the punishment-locked EEG (specifically termed FRN–PE coupling: ρ = 0.43, *p* = 0.003; Figure 3C). None of the other EEG–PE coupling conditions were significantly related to depression or anxiety, and the strong relationship between anxiety and FRN–PE coupling was significantly different than all other correlations (Meng’s *z* > 1.64, *p* < 0.05). Because the relationship between greater anxiety and better punishment learning (Figure 2A) suggests that anxiety influences some aspect of the rate of learning to loss, we examined if FRN–PE coupling predicted learning within the DEP group. The degree of FRN–PE coupling predicted the total learning bias toward NoGo learning (ρ = −0.35, *p* = 0.02), but this effect was mixed between better NoGo learning (ρ = 0.14, *p* = 0.37) and worse Go learning (ρ = −0.37, *p* = 0.01).

### Time–Frequency Plots

**Figure 4.**
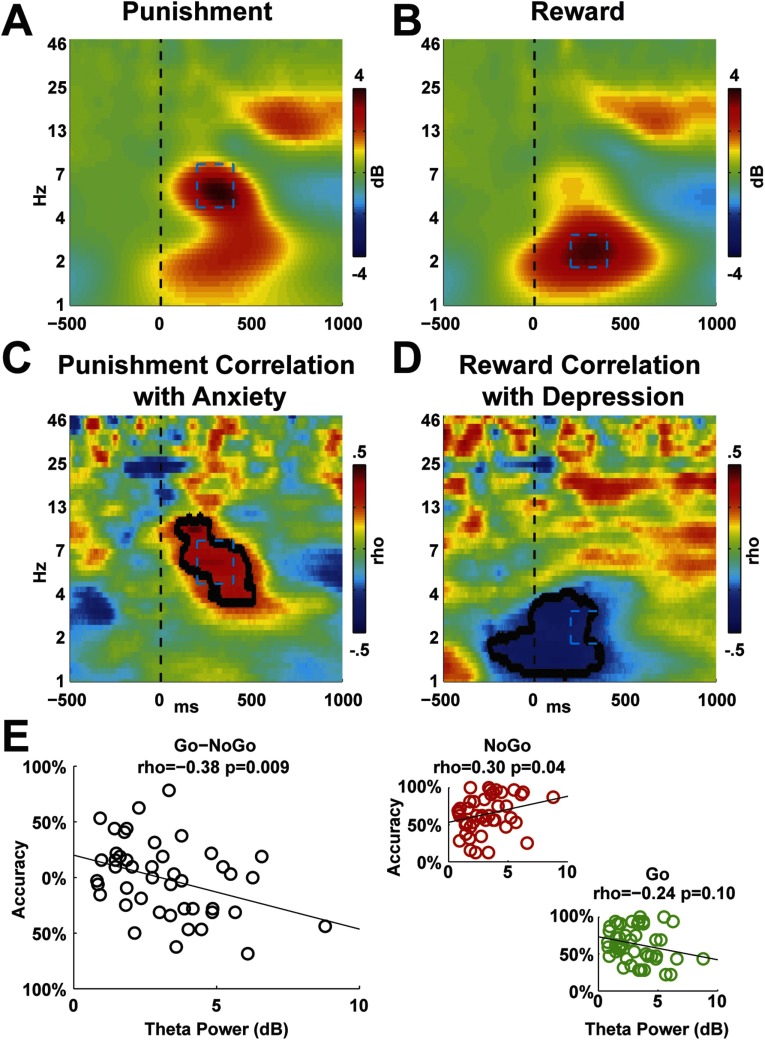
**Time–frequency plots for punishment and reward at electrode FCz.** A–B) Control (CTL) group: punishment led to theta-band activity and reward led to delta-band activity. Tf-ROIs are outlined in cyan boxes. C) DEP group: correlation between punishment power and anxiety. Contours show significant correlations in the same region as the tf-ROI. D) DEP group: correlation between reward power and depression. Contours show significant correlations in a slightly earlier region, yet partially overlapping with the tf-ROI. E) DEP group: correlation between punishment theta power tf-ROI and learning bias (Go–NoGo). This pattern was due to a link between punishment theta and NoGo learning (red), not Go learning (green).

Figure 4 shows the time–frequency plots at electrode FCz. As expected, the punishment condition was characterized by theta-band activity, and the reward condition was characterized by delta-band activity. Figures 4A and 4B show these tf-ROIs defined in the CTL group (the DEP group was identical). Figures 4C and 4D show the correlations within the DEP group between anxiety and depression for these two conditions, respectively. There were no significant correlations between punishment power and depression and reward power and anxiety. The relationship between anxiety and punishment was precisely within the tf-ROI, while the relationship between depression and reward was slightly earlier than expected.

Figure 4E shows the correlation between theta power and Go–NoGo bias in the DEP group (ρ = −0.38, *p* = 0.009), which was due to a significant relationship between punishment theta and NoGo learning (ρ = 0.30, *p* = 0.04) but not Go learning. There were no significant relationships between reward delta power and any learning bias, nor were there any significant correlations within the CTL group. While this trinary relationship between anxiety, punishment theta power, and NoGo learning suggests a mediating effect of punishment theta on the behavioral effect reported in Figure 2, the Sobel test for mediation was nonsignificant (*z* = 1.65).

### Source Estimation

**Figure 5.**
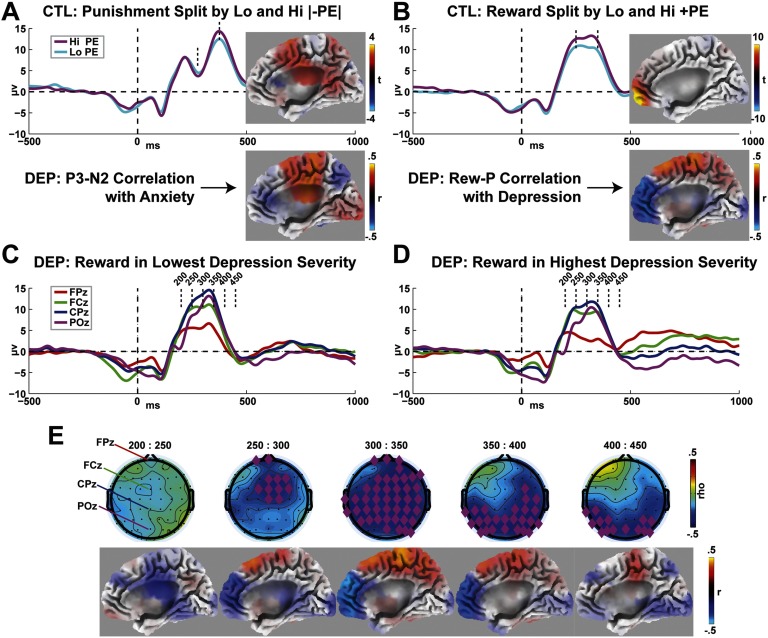
**Source estimation of prediction errors and symptomatology influence.** A) CTL group: the N2–P3 complex was specifically highlighted by the pairwise contrast of high versus low prediction errors; differences in this time window were localized to dorsal cingulate and pre-SMA. In the DEP group, anxiety was correlated with activity in these same areas. B) CTL group: the Rew-P was specifically highlighted by the pairwise contrast of high versus low prediction errors; differences in this time window were localized to orbitofrontal areas. In the DEP group, depression was correlated with activity in this same area. C–D) DEP group: ERPs across the midline from individuals high versus low in depressive symptomatology; the FPz lead (red) displays the largest morphological difference. E) DEP group: scalp maps and source estimations from consecutive 50 ms time windows, showing significant correlations with depressive symptomatology over the time course of the Rew-P. Although the topographic relationships move posteriorly, the major source-estimated influence remains a diminishment in orbitofrontal activity following reward.

Figures 5A and 5B show the splits between high and low PE ERPs and source estimates within the CTL group. In the CTL group, −PE was characterized by increased activity within BAs 24, 32, and 6, extending posteriorly into areas 23 and 31. These align with a depiction of FRN and theta as being generated by dorsal mid-cingulate and supplementary motor areas. In the DEP group, anxiety was positively correlated with activity in these same areas.

In the CTL group, +PE was characterized by increased activation within BAs 10 and 11 (Figure 5B), suggesting an orbitofrontal generator. Within the DEP group, ERPs split between the lowest and highest severity quartiles of the sample (Figures 5C and 5D) suggest two features of the response to reward that are affected by depression: (a) an overall blunting of the Rew-P across the cortex and (b) a specifically notable diminishment in fronto-polar areas. Figure 5E shows how the topography of the Rew-P correlates with depression, with major effects emerging anteriorly within the earliest time window of the Rew-P used here (250–300 ms) and extending to posterior areas over time. Source estimation of the influence of depression across these time windows reveals that these negative topographic correlations are primarily accounted for by specifically diminished activity in BAs 10 and 11, although other broad cortical areas are also differentially involved over time.

### Unique Variance

All correlations with depression or anxiety were repeated with the residualized measures described in the Methods to assess the unique contribution of these predictors. All conclusions described in the Results were unchanged (indeed, most were stronger), with the exception of two small differences. First, the difference in correlations between anxiety and FRN–PE coding and depression and Rew-P–PE coding was nonsignificant. Second, Figure 4C did not reveal any significant correlations between anxiety and theta power in the tf-ROI. We think this first difference is not important, as both independent and dependent variables are different. While the second difference was surprising, it simply reflected a less powerful effect (significant clusters emerged with a cluster size minimum of 200 pixels or at a *p* value threshold of 0.15).

## DISCUSSION

The findings reported herein support the hypothesis that a dimensional account of mood and anxiety disorders is a more effective depiction than a categorical account when interpreting reinforcement-related neural alterations. We previously identified a negativity bias in depressed participants that caused them to overfocus on punishments, leading to better punishment learning (Cavanagh, Bismark et al., 2011). Here we associate that aspect of punishment sensitivity specifically to comorbid anxiety, whereas depressive symptoms were associated with reward-related deficits. In the broadest perspective, both of these categorical and dimensional accounts are accurate, even though they are conflicting. We outline in this section why we think the dimensional account is more important.

Hypersensitivity to punishment is associated with the categorical phenotype of MDD (Beck, 1976; Eshel & Roiser, 2010). Yet, one of the goals of computational psychiatry is to parse heterogeneous phenotypes, which requires both sensitivity and specificity. Here the categorical account fails in specificity. In this report, we identified unique cognitive features that influenced distinct neural processes, sometimes leading to specific behavioral biases. Although admittedly reductionistic, this dimensional approach offers a chance to interpret heterogeneous psychiatric phenotypes as the accumulation of aberrant low-level processes.

The separable influences of comorbid depression and anxiety were dissociated in six important areas tested here: (a) performance, (b) ERP features, (c) information content within ERP features, (d) frequency, (e) spatial generators, and (f) computational role. Following this enumeration, anxiety led to better avoidance learning (a), likely due to a tighter coupling of |−PE| with punishment-specific ERP features (c) and the underlying theta-band dynamics (d) that are generated by dorsal midline premotor structures (e). This account is in line with recent meta-analytic and empirical evidence that anxiety is associated with larger frontal midline theta signals to a variety of signals of the need for control (Cavanagh et al., 2017; Cavanagh & Shackman, 2014; Moser et al., 2013), and these signals are effectively utilized to boost avoidance or caution. This latter point suggests that anxiety boosts the effective computational influence (f) of error signals in that the information content in the signal is utilized to a greater degree.

In contrast, depression led to smaller reward-specific ERP features (c) and underlying delta-band dynamics (d), that appear to be due to orbitofrontal and ventromedial processes (e). This account is in line with suggestions of smaller reward-related EEG signals and orbitofrontal dysfunction in depression (Nelson et al., 2018; Whitton et al., 2016). These reward-related alterations were not used in an “effective” manner, as in anxiety to influence learning, but may reflect an “affective” role of reduced hedonic appreciation within a context of maintained information content (f). This suggests an implied but important dissociation in information content of these signals: Anxiety boosts neural systems involved in avoidance, but depression diminishes neural systems involved in only some aspect of reward responding. Given the observation of intact reward learning, here we suggest that this dysfunction may be hedonic and thus might not be captured by standard reinforcement learning tasks or models.

Whitton et al. (2016) recently provided evidence that diminished Rew-P activity in asymptomatic previously depressed participants was associated with diminished LORETA-resolved perigenual cingulate activity, and this source activity correlated with a deficit in reward learning. There are a number of differences between the implicit learning task used in this study and the task of Pizzagalli used by Whitton et al. One major difference may be that here reward learning is interpreted as a feature of cortico-striatal integration (“ability”), whereas it is interpreted as a feature of hedonic capability (“motivation”) in the latter study. The Rew-P has been shown to be sensitive to affective manipulation (Brown & Cavanagh, 2018; Threadgill & Gable, 2017), so a motivation-specific hypothesis of diminished Rew-P in depression should be easily testable.

### Limitations

We were unable to identify a statistical mediator of the relationship between anxiety and punishment learning, even though theta power was significantly related to both. Although the statistical test of mediation was in line with this hypothesis, it was nonsignificant. Two other outcomes offer some qualifications of the interpretation of these findings. First, the loss learning rate was not related to anxiety. This may be a reflection of the simple model used here: the aim of the model was to better inform the EEG signals and not to provide multiple tests of optimal fitting parameters. Second, while FRN–PE coupling correlated with the overall NoGo–Go bias, it was more strongly related to worse Go learning. This is surprising, but it may simply reflect a loss of specificity in the combination of symptomatology, EEG activity, PE, and two measures of behavioral performance. The coupling between tf-ROIs and PE was also tested;^1^ findings were similar to but weaker than FRN–PE coupling reported following Figure 3. These limitations may be addressed with more specific assessments of the dimensional nature of anxiety.

Source estimation is a strategically useful process for inferring the nature of EEG signals; however, findings should always be interpreted with caution. It is possible that more specific ventral frontal areas are particularly implicated in this depression-related alteration, such as hypoactive subgenual or perigenual cortex, both of which have been implicated in depression (Davidson, Pizzagalli, Nitschke, & Putnam, 2002; Mayberg, 2007). However, the prediction error–specific contrasts used here are ideal for source estimation contrasts, as they reflect pure insertion of a parametric difference (Friston et al., 1996). Better estimation procedures using individual MRIs will boost the spatial specificity of these signals; yet, for a large-amplitude, low-frequency signal like the Rew-P, it is likely that the spatial generators are widely distributed.

### Conclusion

An emerging consensus suggests that biomarkers will be more efficacious at diagnosis and disease classification than an expanded phenotypic characterization (Diaz-Arrastia, Agostini, Madden, & Van Ness, 2009; Gould & Gottesman, 2006; Robbins, Gillan, Smith, de Wit, & Ersche, 2012). EEG is uniquely sensitive to canonical neural operations that underlie emergent psychological constructs (Cavanagh & Castellanos, 2016; Fries, 2009; Siegel, Donner, & Engel, 2012), making it well suited for discovery of aberrant neural mechanisms that underlie complicated disease states (Insel et al., 2010; Montague, Dolan, Friston, & Dayan, 2012). In this report, we provide the first evidence that mood and anxiety dimensions are associated with unique aspects of EEG responses to reward and punishment. This finding compels further research into the domain-specific neural systems underlying dimensional aspects of psychiatric disease (Cuthbert & Insel, 2013).

## AUTHOR CONTRIBUTIONS

James F. Cavanagh: Conceptualization: Lead; Formal analysis: Lead; Writing–original draft: Lead. Andrew W. Bismark: Investigation: Supporting; Writing–review & editing: Supporting. Michael J. Frank: Supervision: Supporting; Writing–review & editing: Supporting. John J. B. Allen: Investigation: Supporting; Methodology: Supporting; Supervision: Supporting; Writing–review & editing: Supporting.

## FUNDING INFORMATION

This work was supported by F31MH082560 and 1P20GM109089-01A1.

## ACKNOWLEDGMENTS

All data and code for this experiment are available on the PRED+CT website, http://www.predictsite.com, accession no. d006.

## Note

^1^ Users can download these data and scripts from http://www.predictsite.com, accession no. d006.
